# A Relevance Vector Machine-Based Approach with Application to Oil Sand Pump Prognostics

**DOI:** 10.3390/s130912663

**Published:** 2013-09-18

**Authors:** Jinfei Hu, Peter W. Tse

**Affiliations:** The Smart Engineering Asset Management Laboratory (SEAM), Department of Systems Engineering and Engineering Management (SEEM), City University of Hong Kong, Tat Chee Avenue, Kowloon, Hong Kong 999077, China; E-Mail: jinfeihu-c@my.cityu.edu.hk

**Keywords:** pump impeller, remaining useful life (RUL), prognosis, relevance vector machine (RVM), sum of two exponential functions

## Abstract

Oil sand pumps are widely used in the mining industry for the delivery of mixtures of abrasive solids and liquids. Because they operate under highly adverse conditions, these pumps usually experience significant wear. Consequently, equipment owners are quite often forced to invest substantially in system maintenance to avoid unscheduled downtime. In this study, an approach combining relevance vector machines (RVMs) with a sum of two exponential functions was developed to predict the remaining useful life (RUL) of field pump impellers. To handle field vibration data, a novel feature extracting process was proposed to arrive at a feature varying with the development of damage in the pump impellers. A case study involving two field datasets demonstrated the effectiveness of the developed method. Compared with standalone exponential fitting, the proposed RVM-based model was much better able to predict the remaining useful life of pump impellers.

## 1. Introduction

Slurry pumps are widely used to remove mixtures of abrasive solids and liquids in wet mineral processing operations. These pumps usually experience severe erosive and/or corrosive wear even under normal working conditions. Consequently, their performance becomes severely comprised over time and at a certain point, the pumps will begin to fail without warning. The prevention of such unscheduled downtime requires substantial investment to maintain the system near the initially intended maximum level of efficiency. In particular, it becomes necessary to implement a scheduled preventive maintenance program capable of predicting the trend of degradation and estimating the remaining useful life of the pumps, to ensure a safe, economical, and efficient operation of the pump systems in the field. The remaining useful life of an asset or system is defined as the length of time from the present time to the end of the asset's useful life [1].

A review of the literature on the degradation of slurry pumps shows that several studies have investigated the process of wear associated with the machines and the possibility of improving pump performance through the use of more durable materials [2–6]. Llewellyn *et al.* applied the Coriolis method to assess the scouring attack resistance of cast alloys for slurry pump components [3]. Their tests were useful in evaluating the scouring erosion resistance of metallic, ceramic, and cement materials for various slurry transport components. Walker [4] compared the field wear performance of side-liners with that anticipated from laboratory data. The results showed that the field wear patterns observed were generally similar to the wear patterns predicted from laboratory data. However, the field wear involved a particle size whose effect on the white iron components of the machines was milder than that seen in the laboratory tests, which were based on grey iron. This difference might be explained by the difference in the levels of hardness between the two types of parts. Dong *et al.* [5] applied a liquid-solid two-phase flow theory to study the wear location and the process of centrifugal slurry pump working for dense medium cyclone coal preparation. They mainly analyzed the wear location on the pump impeller and the factors that influenced wear, such as the diameter, velocity, shape, and invasion angle of the coal particles.

However, compared with the studies on wear, few research articles have been devoted to the problem of monitoring the condition of slurry pumps, and even fewer have reported on the issue of fault diagnosis in slurry pumps [7–11]. Zhao *et al.* [7] modified the neighborhood rough set model by setting different neighborhood sizes for different features and used this model for feature selection in the vibration-based fault diagnosis of slurry pump impellers. The data collected from laboratory experiments showed that the use of the features identified by the model proposed by Zhao *et al.* could yield much greater accuracy in classification. Qu and Zuo [8] proposed a data processing algorithm to clean the data based on support vector classification and random sub-sampling validation. A sequential backward selection method was used to identify irrelevant features. The method performed well in relation to laboratory pump data. Hancock and Zhang [9] developed an online hydraulic vane pump fault detection system. In this system, pump vibration signals were decomposed using a wavelet packet analysis, and an adaptive neuro-fuzzy inference system was used to distinguish between functioning and failed pumps. Maio *et al.* [10] proposed an ensemble approach comprising fuzzy C-means and hierarchical trees to assess the wear status of oil sand pumps. This method performed well in terms of diagnosis when it was evaluated on the basis of data collected from oil sand pumps in the field. It should be noted that all of the research cited above has been limited to the problem of fault diagnosis in slurry pumps through the use of classification methods. In particular, although the problem has received some attention in other contexts [12–15], few studies have addressed the issue of “prognostics” that is arguably the most important part of the condition-based maintenance of slurry pumps. Due to its capacity for prior event analysis, prognosis is more effective than diagnostics in assuring a zero-downtime performance of the machinery. In general, health prognosis involves three main steps: evaluating the machinery's current condition [16], observing its future condition, and predicting the residual useful life of the equipment before the failures eventually occur [17].

The research reported in this paper was conducted in response to a particular requirement in oil mining whereby slurry pumps need enhanced monitoring because they are prone to sporadic catastrophic breakdowns. In the oil-mining sector, equipment owners need to be aware when their pumps require an overhaul or when the related pump components will shortly need to be replaced to avoid unplanned pump downtime. To reduce potential costs, it is of great practical importance to have available a method to monitor the condition of the pump that is capable of determining when it should be overhauled or replaced, or how long its useful life is expected to be. Experience has shown that slurry pumps wear mainly because of impeller failure that can be indicated in advance by a decrease in impeller diameter [2]. This suggests that the impeller might be used as the target of monitoring to assess the health of the pump and to calculate the associated estimation of remaining useful life. The reliable prediction of the remaining useful life (RUL) of pumps is likely to yield considerable cost savings and improvements in operational safety.

Recent years have witnessed the rapid development of RUL prediction methods for maintenance [1]. Many RUL prediction approaches have been proposed and they can be broadly categorized into: physics-of-failure approaches [18–20], data-driven approaches [1,21,22], and fusion approaches [23,24]. Physics-based models rely on the understanding of physics-of-failure mechanisms. By conducting physics-of-failure experiments, Jin *et al.* identified the failure mechanism of lubricant loss in a space bearing, and on this basis proposed a physics-of-failure-based degradation model and life prediction method for the Momentum Wheel in long-life satellites [19]. However, it is typically difficult to understand the physical failure mechanisms in complex engineered systems that generally consist of multiple components. Furthermore, it is too expensive and time-consuming to test systems to physical failure through experimentation [23]. Data-driven approaches derive models directly or indirectly from condition-based data collection. With the rapid development of technologies for the acquisition, storage, and processing of data, such data-driven approaches have become widely used. Si *et al.* presented an excellent review of data-driven prognostic approaches associated with the estimation of RUL [1]. They pointed out that many challenges remained for further study, especially in relation to some practical engineering-oriented problems. The fusion method combines the physics-of-failure and the data-driven models and hence combines some of the merits of these two approaches. Cheng and Pecht proposed a fusion prognostic method to predict the remaining useful life of electronic products [25]. In their approach, the physics-of-failure model was used to identify the products' failure mechanisms, failure models, and critical parameters, and the data-driven method was used to obtain the indicators and to detect the state of the monitored product.

As the data were sampled from oil sand pumps under extremely complicated and adverse working conditions, which may include all kinds of disturbances, the use of data pre-processing, feature extracting, and model building in this study was much more challenging than in research based on laboratory datasets. To the best of our knowledge, little effort has been devoted to pump prognosis based on field datasets in the literature. In this study, relevance vector machines (RVMs) were combined with the sum of two exponential functions to arrive at a method capable of predicting the degree of wear of field impellers and their remaining useful life. RVM—a method first introduced by Tipping [26]—is a data-driven method with the Bayesian treatment of the support vector machine (SVM). Hence, in contrast to the SVM, the RVM naturally incorporates prior knowledge. The key feature of an RVM is that it is an order of magnitude more compact than the corresponding SVM [27]. This compactness results in a significant improvement in the process speed while offering a generalization performance through sparse predictors comparable to an SVM. Additionally, an RVM represents a mechanism that can avoid over-fitting by implementing a priori knowledge on the model weights. The RVM has been proven to be an efficient prognostic technique in many applications [24,28–30]. Saha *et al.* developed a fusion method by combining relevance vector machines (RVMs) and particle filters to predict the RUL of a lithium-ion battery [24]. Their well-developed fusion method was based on knowledge of the physics-of-failure model of the battery. Zio and Di Maio combined RVMs with a Paris-Erdogan growth function to describe the health deterioration of a fatigue crack growth process [29]. Di Maio *et al.* explored the use of a combination of RVMs and one exponential function to predict the RUL of partially degraded thrust ball bearings [30]. Their method showed good RUL estimation accuracy and the capability of uncertainty while directly handling the vibration signals of bearings. Wang *et al.* combined RVMs and a conditional three-parameter function to predict the remaining useful life of lithium-ion batteries [32]. In this study, the sum of exponential functions is chosen due to the much flexibility to fit the very complex degradation curves. The historical data on the pump impeller in question were first used to extract some useful feature(s). The RVM-based model was then trained by an input vector constructed by a serial of inspection file numbers and the target vector was indirectly obtained from these feature(s) so that the corresponding degradation evolution curve could be calculated. Finally, the pump's remaining useful life was estimated by extrapolating the degradation evolution curve up to the predefined alert threshold.

The remainder of the paper is organized as follows: after an introduction to the basic theory of RVM in Section 2, Section 3 presents a prediction of the deterioration trend and RUL in a field oil sand pump derived from vibration-based degradation signals. In Section 4, the results of this prognostic procedure are presented and the prognostic performance of the developed model applied to real data is analyzed. We conclude the paper in Section 5.

## 2. Introduction to RVM

An RVM is a Bayesian sparse kernel model that introduces a prior distribution over the model weights that are governed by a set of hyper-parameters [33]. In comparison with the equivalent SVM, the most compelling feature of the RVM is its superior generalization performance and a shorter time for prediction because relatively few “relevance vectors” are used in effecting the prediction [34]. The RVM also provides posterior probabilistic outputs. Taking these advantages into consideration, in this study an RVM is adopted to build a degradation model to predict the remaining useful life of the machine components.

The RVM starts with the concept of linear regression models that are generally used to find the parameter vector **w**={*w*_0_,*w*_1_,*w*_2_,…,*w_N_*} For a new input **x** (**x** ∈ ℜ*^N^*), the prediction of **z** can be obtained according to the following equation:

(1) $$\text{z}=\Phi \text{w}+\varepsilon_{n}$$

where **Φ** is a *N* ×(*N*+1) design matrix, constructed with the *i^th^* row vector denoted by **Φ***_i_*(*x_n_*)=[1,*K*(*x_n_*,*x*_1_),*K*(*x_n_*,*x*_2_),…,*K*(*x_n_*,*x_N_*)]; the offset *ε_n_* is an additional noise component of the measurement with mean zero and variance *σ*^2^ In this way, the likelihood of the dataset can be written as:

(2) $$p(\text{z}\;|\;\text{w},\sigma^{2})={\left(2\pi \sigma^{2}\right)}^{N-2}\exp\{-\frac{1}{2\sigma^{2}}\left\|\text{z}-\Phi \text{w}\right\|\}$$

In many applications, due to the singularity of the coefficient matrix in Equation (1), over-fitting problems may arise during the maximum likelihood estimation of parameters in Equation (2). This could lead to poor prediction performance. To overcome this problem, Tipping proposed the use of additional constraints on the parameter vector, **w**[26].

In the RVM learning process, the parameter vector **w** is constrained by putting a zero mean Gaussian prior distribution on the weights, that is:

(3) $$p(\text{w}|\alpha )=\prod_{i=1}^{\text{M}}N({\text{w}}_{i}|0,\alpha_{i}^{-1}),$$

where *α_i_* is used to describe the inverse variance of each vector w*_i_*, and **α** denotes as (*α*_1_,*α*_2_,…,*α_M_*). From this formulation, it can be easily seen that there is an individual hyper-parameter *α_i_* associated with each weight to control how far each parameter vector is allowed to deviate from zero [33].

By Bayes' rule, the posterior probability over all of the unknown parameters can be expressed as:

(4) $$p(\text{w},\alpha ,\sigma^{2}|\text{z})=\frac{p(\text{z}|\text{w},\alpha ,\sigma^{2})\;p(\text{w},\alpha ,\sigma^{2})}{p(\text{z})},$$

where:

(5) $$p(\text{z})=∭p(\text{z}|\text{w},\alpha ,\sigma^{2})\;p(\text{w},\alpha ,\sigma^{2})dw\mathrm{d;}\alpha d\sigma^{2}.$$

However, the solution of the posterior *P*(**w,α**, *σ*^2^| **z**) in Equation (4) cannot be computed directly because the normalizing integral on Equation (5) cannot to be executed. Instead, we decompose the posterior as:

(6) $$p(\text{w},\alpha ,\sigma^{2}|\text{z})=p(\text{w}|\alpha ,\sigma^{2},\text{z})\;p(\alpha ,\sigma^{2}|\text{z}).$$

According to Bayes' rule, the posterior distribution over weights can be expressed as:

(7) $$p(\text{w}|\alpha ,\sigma^{2},z)=\frac{p(\text{z}|\text{w},\sigma^{2})\;p(\text{w}|\alpha )}{p(\text{z}|\alpha ,\sigma^{2})}∼N(\text{m},\sum ),$$

where the mean **m** and covariance **Σ** are:

(8) $$\text{m}=\sigma^{-2}\sum \Phi^{T}\text{z},$$

(9) $$\sum ={\left(\text{A}+\sigma^{-2}\Phi^{T}\Phi \right)}^{-1},$$

where **A** = *diag*(**α**)= *diag(α*_0_,*α*_1_,…,*α_N_*).

The probability distribution over the training targets can be obtained by integrating the weights to obtain the marginal likelihood for the hyper-parameters:

(10) $$p(\text{z}|\alpha ,\sigma^{2})=\int p(\text{z}|\text{w},\sigma^{2})p(\text{w}|\alpha )d\text{w}∼\text{N}(0,\text{C}\text{)},)$$

where the covariance matrix is given by **C** =*σ*^−2^**I**+ **ΦA**^−1^**Φ***^T^*. Then the log probability distribution over the training targets is:

(11) $$\ln p(\text{z}|\alpha ,\sigma^{2})=\frac{N}{2}\text{ln}(\sigma^{-2})-\frac{1}{2}(\sigma^{-2}{\text{z}}^{T}\text{z}-{\text{m}}^{T}\sum^{-1}\text{m})-\frac{N}{2}\ln(2\pi )+\frac{1}{2}\sum_{i=0}^{N}\text{ln}(\alpha_{i}).$$

Thus, the estimated value of the parameter weights **w** is given by the mean of the posterior distribution in Equation (7), and the hyper-parameters **α** and *σ*^2^ can be estimated by maximizing Equation (11), which is known as the evidence approximation procedure. Further details on the approximation procedure are available at [33]. For a new input, *x_new_*, the probability distribution of the predictor z*_new_* is given by:

(12) $$p({\text{z}}_{\text{new}}|x_{\text{new}},\hat{\alpha },{\hat{\sigma }}^{2})=\int p({\text{z}}_{\text{new}}|x_{\text{new}},\text{w},{\hat{\sigma }}^{2})p(\text{w}|\text{z},\hat{\alpha },{\hat{\sigma }}^{2})d\text{w}∼N(m_{\text{new}},\sigma_{\text{new}}^{2}),$$

where the mean and variance of the predictor are:

(13) $$m_{\text{new}}={\text{m}}^{T}\Phi (x_{\text{new}}),$$

and:

(14) $$\sigma_{\text{new}}^{2}={\hat{\sigma }}^{2}+\Phi {\left(x_{\text{new}}\right)}^{T}\sum \Phi (x_{\text{new}}),$$

respectively.

## 3. Application of the Model to the Oil Sand Pump

**Figure 1.**
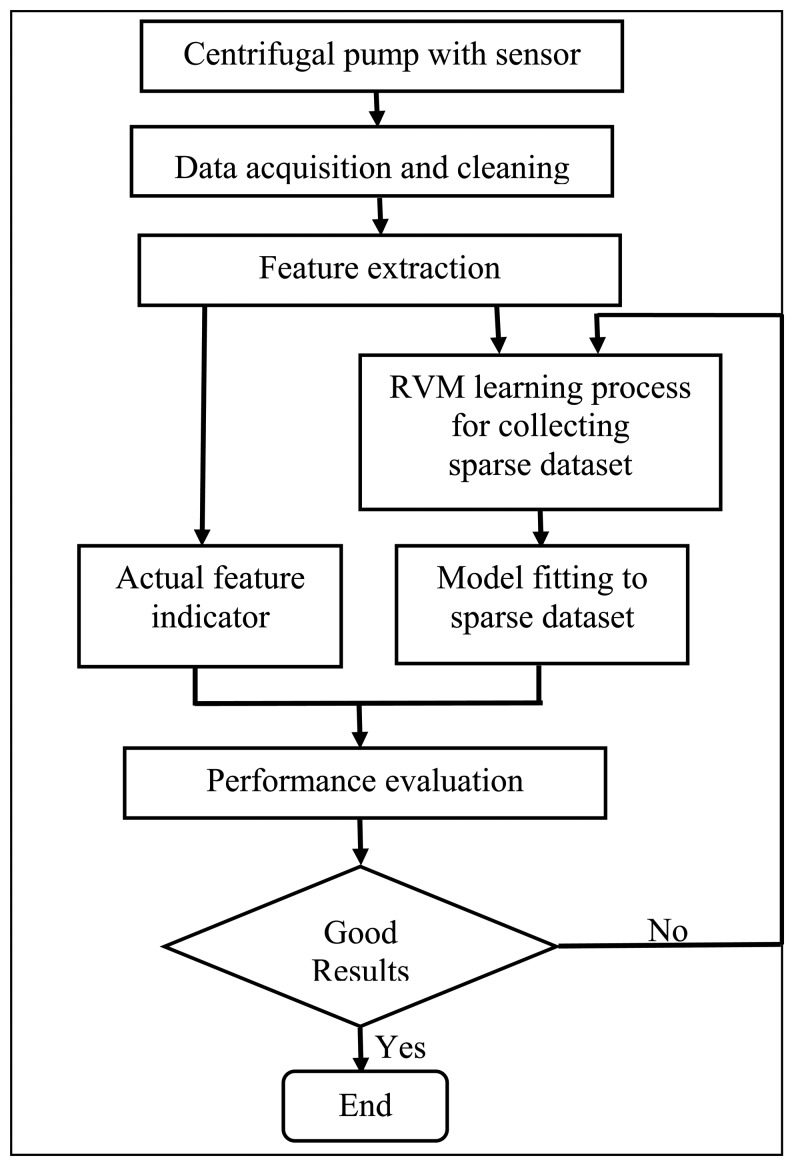
Schematic diagram of the developed method.

Slurry pumps are used to deliver a mixture of bitumen, sand, and small pieces of rock from one site to another in wet mineral processing operations. Experience has shown that the components of slurry pumps undergo a great variety and degree of abrasiveness and erosion. Often, the pump wear results in sudden downtime. This leads to huge economic losses due to the interruption of the mineral processing operations. Hence, it is of critical significance to have a method that is capable of helping to decide when a pump should be taken out of service and overhauled. In this study, a prognostic method is developed to assess the pump's performance degradation and to predict the RUL of the pump. The schematic diagram of the developed method is depicted in Figure 1. The method involves three steps: data acquisition and feature extraction, sparse dataset acquisition through the RVM learning process, and model fitting and prediction by extrapolating the fitted model. Further details about each step are given in the following subsections.

### 3.1. Data Collection

**Figure 2.**
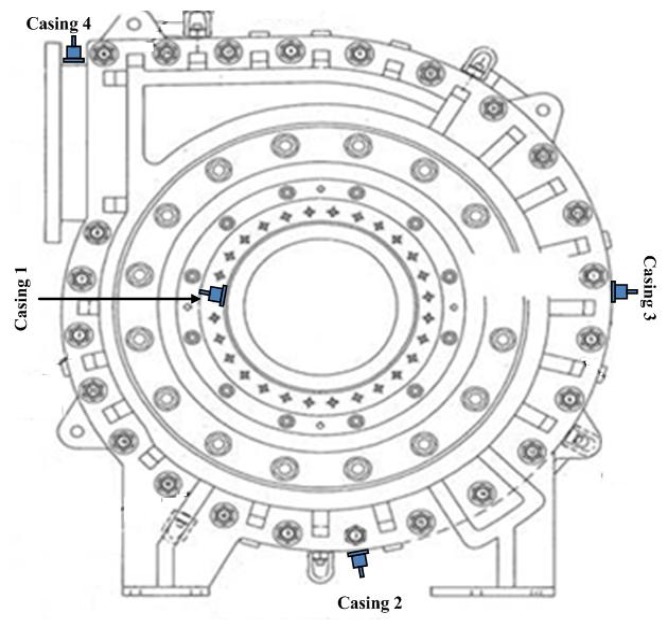
The measurement locations of the four accelerometers mounted on a slurry pump (the four accelerometers named as casing 1, 2, 3 and 4) [35].

Field data were collected from the inlet and outlet of slurry pumps operating in an oil sand mine. Vibration signals using the same sampling frequency rate (51.2 kHz) were obtained from four accelerometers mounted at four different pump locations. These four accelerometers were named as casing 1, casing 2, casing 3, and casing 4, respectively, in Figure 2. Data collection began immediately after all of the components inside the pump had been renewed. It was continued intermittently for around three months with one sampling per hour until the pump's impeller wore out sufficiently to need replacement. In total, the pump was subjected to 904 measurement hours. The increased vibration levels in certain components of the pump indicated the level of degradation of the pumps, so the vibration signals could be used to monitor the health of the pump system. Data cleaning was done by manually removing outliers exceeding a predefined threshold.

### 3.2. Characteristic Frequency of the Pump

**Figure 3.**
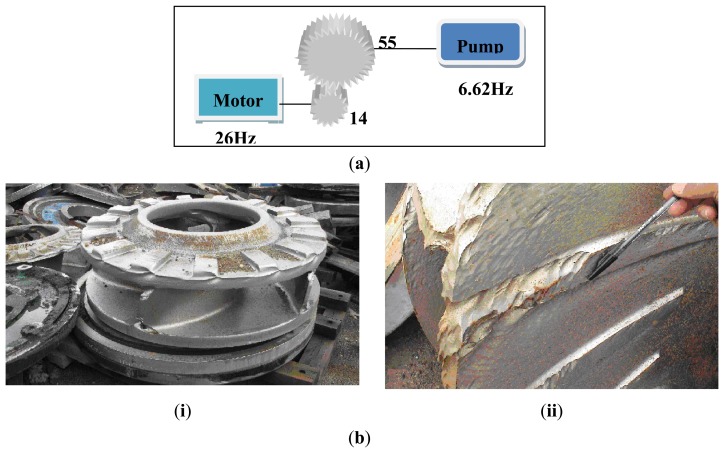
(**a**) The pump speed stepped down through a gear box; (**b**) The damages caused to the impeller's vanes by oil sand and small rocks (**i**) The impeller with damaged vanes; (**ii**) The close up view of one of the damaged vanes.

The fast Fourier transform (FFT) technique converts time-domain signals into frequency-domain signals and can thereby identify salient features in machines [36]. In this application, the characteristic frequencies of the oil sand pumps were analyzed using FFT. As shown in Figure 3a, the motor on the pump used at the oil sand mine generally ran at 1,526 rpm (26 Hz), and was stepped down through a gearbox to drive the pump at a speed of 388.4 rpm (6.62 Hz). The pumps usually showed quite strong vibration components at 1× its shaft rotational frequency, known as the pump rotating frequency at 6.62 Hz. The impeller's vane-passing frequency was 4× its shaft rotational frequency [37]. Because the impeller used in this study had four vanes, therefore, the vane-passing frequency was 26.48 Hz. The first harmonic frequency and the second harmonic frequency of the tooth meshing frequency were calculated from experimental data as 364 Hz and 728 Hz respectively. Figure 3b(i) showed the damages caused to the impeller's vanes by oil sand and small rocks and Figure 3b(ii) showed the close up view of one of the damaged vanes. These pictures were captured in the oil sand exploration field. One can see that the damages to the vanes caused by the bombardment of oil sands are severe and cannot be comparable to those caused by normal water and oil pumps.

**Figure 4.**
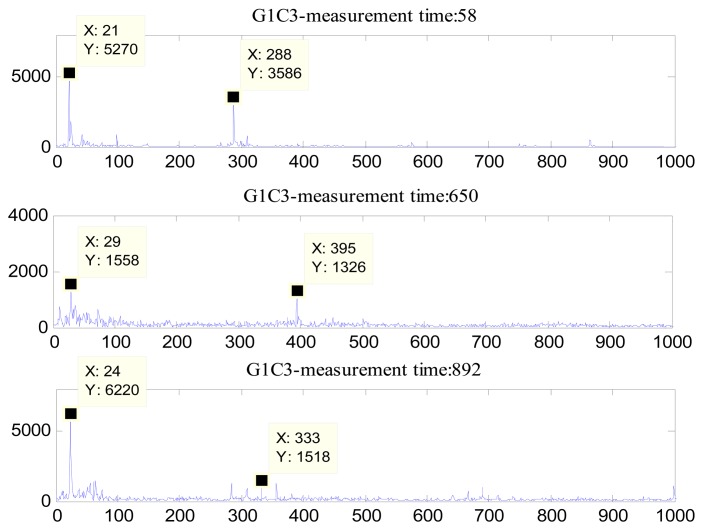
The frequency spectra of the vibrations collected from the oil sand pump.

Three representative frequency spectrums of the oil sand pump are shown in Figure 4. It is clearly seen that none of the frequencies (the motor frequency, the vane-passing frequency, or the tooth meshing frequency) were exactly equal to the characteristic frequencies. The reason is that in practical installations, the working motor speed often fluctuates or drifts. Therefore, in the subsequent step of feature extraction, a narrow spectrum band was selected within which an averaging algorithm was executed and the corresponding results were used to substitute for the vane-passing frequency.

### 3.3. Feature Extraction

**Figure 5.**
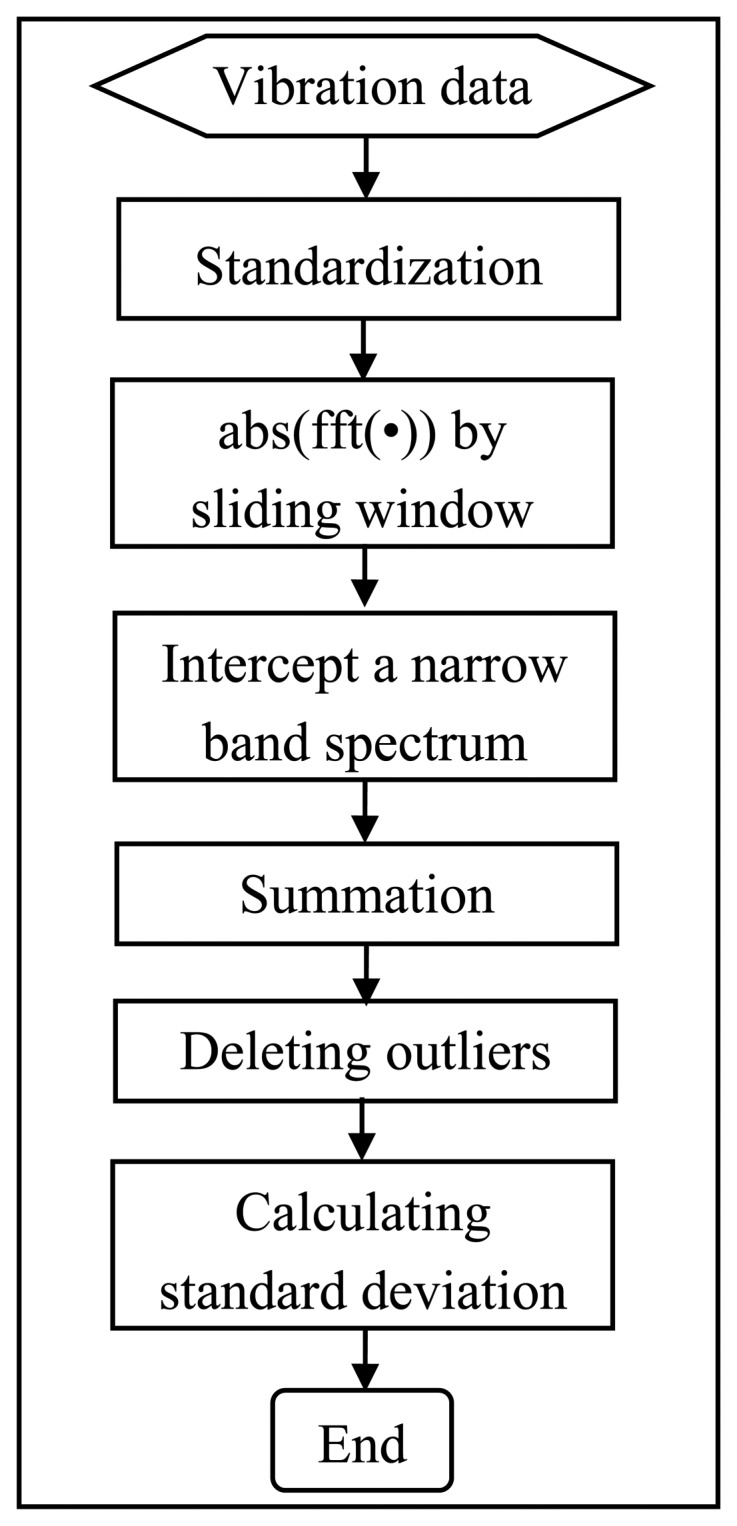
Feature extraction process.

In the mineral processing field, some critical components of slurry pumps frequently fail earlier than their expected service time. For example, according to field observations, the vanes of impellers were usually the first component to wear out due to abrasion from the fluid-solid materials [11]. This research therefore focuses on the pump impeller. Instead of using the crude vibration data directly for the prognosis of the pumps' health, a feature extraction procedure was implemented to identify feature(s) that indicated the clear progressive degradation of the pump impeller. As shown in Figure 5, a suite of processes was designed to extract an indicator that would vary increasingly with progressive damage to the pump.

The details of the feature extraction process are given as follows. At the first step, the vibration data **X**(*T*,*n*) were standardized to scale all of the data into the same interval, thus:

(15) $${\text{X}}_{\text{new}}(T,n)=\frac{\text{X}(T,n)-\text{mean}(\text{X}(T,n))}{\text{std}(\text{X}(T,n))},$$

where **X**_new_(*T*,*n*) is the standardized data; mean(**X**(*T*,*n*)) is the mean value of the elements in vector **X**(*T*,*n*); std(**X**(*T*,*n*)) returns the standard deviation; *T* is the pump measurement time and *T* =1,2,…,904, *n* is the sample number index for each *T* (*n* = 1,2,…,*N* and N = 51,200).

A Fourier transform-based sliding-window averaging technique was then used to obtain averaged FFT amplitude values **Y**(*t*, *f*) by sliding a window along a sequence of pump-measurement times, thus:

(16) $$Y(T,f\text{)}=\frac{1}{L}\sum_{T=l}^{l+L}\left|\sum_{n=0}^{N-1}{\text{X}}_{\text{new}}(T,n)\exp(\frac{-i2\pi \;fn}{N})\right|$$

where **Y**(*t*, *f*) is the averaged FFT amplitude value; *f* is the frequency index and *L* is the window width.

Then, the averaged FFT amplitude values **Y**(*t*, *f*) were summed up within a narrow spectrum band, 19∼40 Hz and the energy **V**(*T*) was calculated by integrating the frequency within the narrow spectrum band:

(17) $$V(T\text{)}=\sum_{f=19}^{40}Y(T,f\text{)},$$

where the energy **V**(*T*) is taken into account to substitute for the “rating frequency” of the vane-passing frequency of the monitoring pump. The frequency band was selected from the overall frequency band by manually checking the frequency bands of all of the pump-measurement times one by one to ensure that all of the situations had been included.

Finally, the sequential standard deviation values *STD*(*j*) were calculated by augmenting one element from the cleaned summed results, thus:

(18) STD(j)=std(V(1),V(2),…,V(j+q−1)).

where *j* is referred to the file number index and *j* = 1,2,…,*K* − *q* +1, and *q* indicates the file numbers at the steady stage. *STD*(1) was calculated from the first *q* elements that were regarded as the steady stage as impeller deterioration progresses.

**Figure 6.**
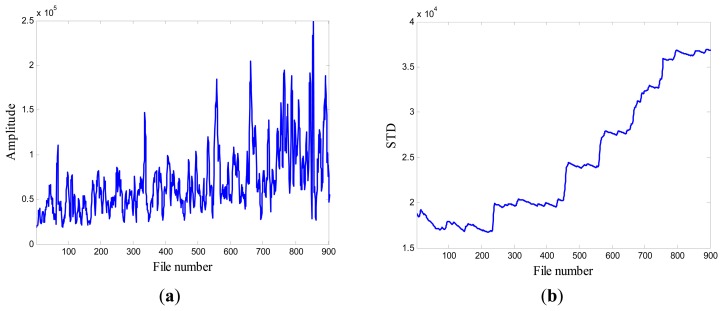
(**a**) Energy evolution (T2G1C3); (**b**) The standard deviation values (T2G1C3).

When compared with the progression of pump damage demonstrated by the energy evolution based on averaged FFT amplitude as shown in Figure 6, the standard deviation contains similar information on the condition of the pump's health. Furthermore, it illustrates a progressive trend of developing damage along the file number. For this reason, this feature was selected as the favorite and most effective candidate to be a feature to monitor the health of the pump.

### 3.4. RVM Learning Process and Model Fitting

**Figure 7.**
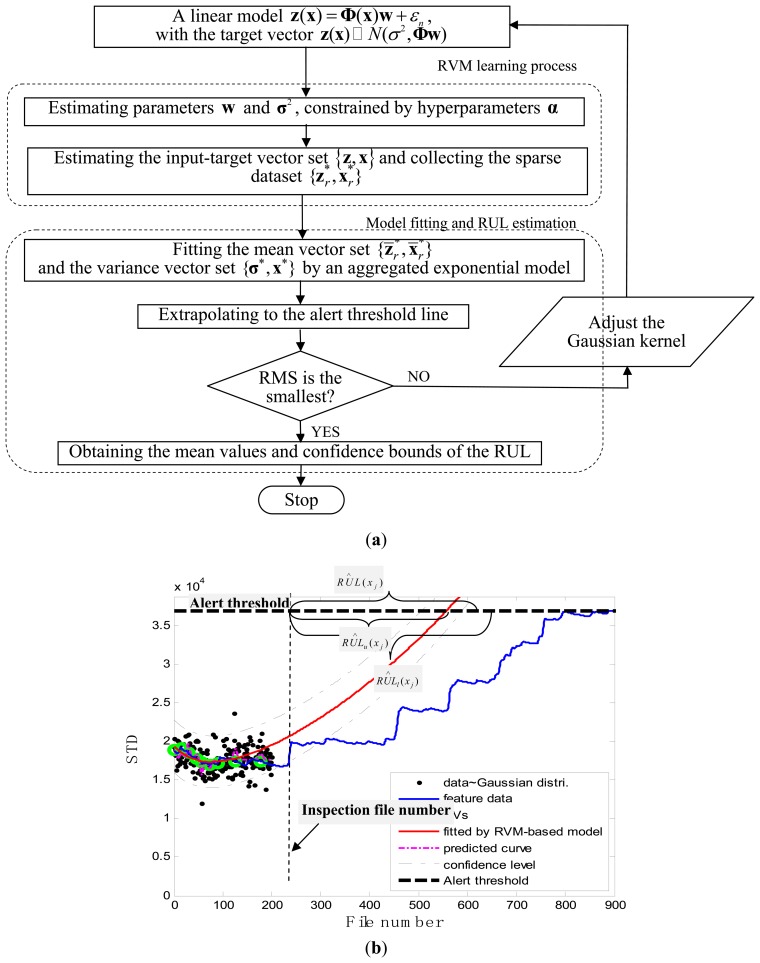
RUL estimation process (**a**) Flowchart (**b**) The mean values and the corresponding confidence bounds of the estimated remaining useful life.

The RVM learning process was performed on the pair of vectors {**z**,**x**}, where the input vector **x** was constructed from successive inspection file numbers. The target vector **z** was constructed by generating the corresponding random numbers that follow the Gaussian distribution with mean values equal to a serial of STD values and variance values equal to a certain pre-defined value. The detailed flowchart for the RUL estimation is shown in Figure 7a. At each inspection file number *x_j_*, *j* = 1,…,*j*, the target values **z** = {*z*_1_,*z*_2_,…,*z_j_*} indicating the pump degradation information were assumed to be known up to *x_j_*. To train the RVM model, a Gaussian kernel was used as the mapping feature space and the value of kernel width was determined using a one-dimensional search method from 30 to 80 with a step length of 0.5 with a view to obtaining the optimized RVM training process with the smallest root mean square error (RMS). Thus the hyper-parameters **w** and *σ*^2^ in Equation (1) were determined during the machine learning process. After building the RVM training model, the representative estimators ${\text{z}}_{r}^{*}=\{{\text{z}}_{1}^{*},{\text{z}}_{2}^{*},\ldots ,{\text{z}}_{r}^{*}\}$ (upon renumbering) whose number was much smaller than that of the training data, were found at the corresponding inspection file numbers ${\text{x}}_{r}^{*}=\{x_{1}^{*},x_{2}^{*},\ldots ,x_{r}^{*}\}$ (upon renumbering), denoted as a sparse dataset $\left\{{\text{z}}_{r}^{*},{\text{x}}_{r}^{*}\right\}$. The pairs of feature data $\left\{{\text{STD}}_{r}^{*},{\text{x}}_{r}^{*}\right\}$ associated with the sparse dataset were labeled as Relevance Vectors (RVs) [33].

An exponential function, a polynomial function and a sum of two exponential functions were the potential candidates to approximate the pump degradation curve. The reasons why we chose the sum of two exponential functions were given as follows. First, compared with the exponential function and the polynomial function with a low degree, the sum of exponential functions was more flexible to fit a complex degradation curve, which had been proven in reference [38]. Second, even though the polynomial function with a high degree showed a flexible fitting characteristic, the function becomes more complex as the order increases. The posterior estimation of the parameters used in the polynomial function with a high degree becomes extremely difficult. The goodness of fit statistics was used to quantify the performance of different functions. The *R*-square, the adjusted *R*-square and the root mean squared error (RMSE) were tabulated in Table 1, where the three statistical values demonstrated that the sum of two exponential functions was better than the exponential function and the Quadratic polynomial (a polynomial function with a degree of two) to fit the pump degradation curve. Therefore, considering the above two reasons, we made a trade-off between the flexibility and the complexity and chose the sum of two exponential functions.

Hence in this study, the sum of two exponential functions was used to fit the degradation evolution of the pump impeller on the basis of the vector constructed by the mean values of the sparse dataset, referred to as $\left\{{\bar{\text{z}}}_{r}^{*},{\bar{\text{x}}}_{r}^{*}\right\}$, where ${\bar{\text{z}}}_{r}^{*}=\{{\bar{\text{z}}}_{1}^{*},{\bar{\text{z}}}_{2}^{*},\ldots ,{\bar{\text{z}}}_{r}^{*}\}$, ${\bar{\text{x}}}_{r}^{*}={\text{x}}_{r}^{*}$. It is derived as follows:

(19) $${\bar{\text{z}}}_{i}^{*}(x_{i}^{*})=a\cdot \exp(b\cdot x_{i}^{*})+c\cdot \exp(d\cdot x_{i}^{*}),\text{i}=1,2,\ldots ,\text{r},$$

where the parameters *a*, *b*, *c* and *d* are the fitted coefficients.

The future evolution of degradation was predicted by extrapolating the fitted model along the inspection file number and the degradation trajectories were traced up to a pre-defined failure threshold; thus simultaneously the mean values of the remaining useful life (RUL) were obtained. The corresponding point *T_j_*, at which the alert threshold line and the fitted degradation curve intersect, is derived as:

(20) $$T_{j}=\frac{\log{\text{z}}_{F}-\log a-\log c}{b+d},$$

where *z_F_* is the predefined alert threshold value. Hence, the estimated mean value of the remaining useful life (RUL) at the inspection file number *x_j_* is calculated as:

(21) $$R\hat{U}L(x_{j})=T_{j}-x_{j}.$$

The correspondence variance vector, $\sigma^{*}=\{\sigma_{1}^{*},\sigma_{2}^{*},\ldots ,\sigma_{r}^{*}\}$ of the predictors associated with the selected RVs can be calculated by Equations (13) and (14). Then the sum of two exponential functions was used to fit the RUL confidence interval curves based on the vector {σ*,x*} The future evolution of RUL confidence interval was predicted by extrapolating the fitted model for the RUL confidence interval along the inspection file number. In this study, the lower and upper RUL confidence bounds *RÛL_l_*(*x_j_*) and *RÛL_u_*(*x_j_*) were estimated by the “two sigma” rule, *i.e.*, a 95.45% confidence level.

Figure 7b illustrates the prognostic process and the corresponding results. In the figure, the black spots represent the target data **z***_j_*, the green circles are RVs deemed to be representative of the evolution of the degradation of the impeller, the thick black dashed line is the alert threshold line, and the purple dashed line is the regression line approximating the real degradation curve. The estimated remaining useful life *RÛL*(*x_j_*) at the inspection file number *x_j_* was obtained by projecting the degradation trajectories (starting from the inspection file number *x_j_*) into the alert threshold (ending with the intersected point). The thin black dashed lines in Figure 7b are the confidence interval lines for the estimated RUL. It is worth noting that at the oil sand mining site, otherwise well-functioning pumps are often forced to stop to replace the components before they are absolutely out of service so as to preempt production shutdowns. Consequently, the precise failure threshold for the impeller cannot be obtained from the field data. In conventional practice, the failure thresholds are set by the users on the basis of heuristically determined safe operational limits. In contrast, in this study, instead of failure thresholds, alert thresholds for pump impellers, beyond which alarms of the pump health are issued and the pump impellers may fail, were set on the basis of our empirical model and pump degradation trend.

## 4. Results and Prognostic Performance Analysis

The application of the prognostic procedure to the calculation of the estimated *RÛL*(*x_j_*) at the inspection file number *x_j_* of the impeller is hereafter illustrated using two datasets sampled from different positions on the same pump. These datasets are referred to as **T2G1C3** and **T2G1C4**. When verifying the RVM-based model on the basis of the empirical data, it is assumed that the equipment may start to fail beyond the maximum degradation level. The performance of the developed procedure for estimating the impeller RUL is evaluated by comparing the results obtained by the RVM-based model and the conventional exponential fitting.

### Case 1: T2G1C3

**Figure 8.**
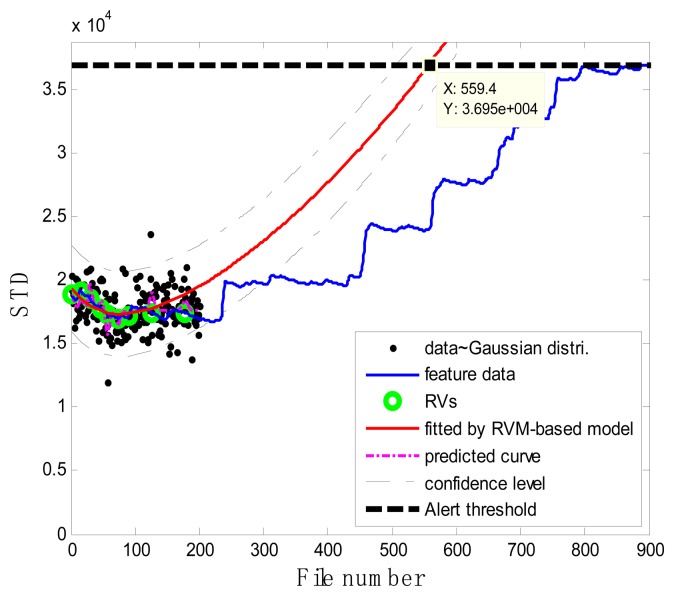
Inspection file number *x*_200_ = 200.

**Figure 9.**
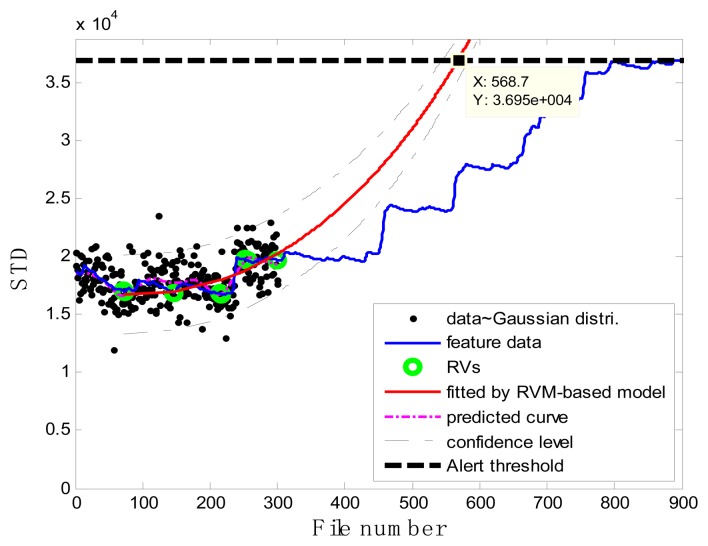
Inspection file number *x*_300_ = 300.

**Figure 10.**
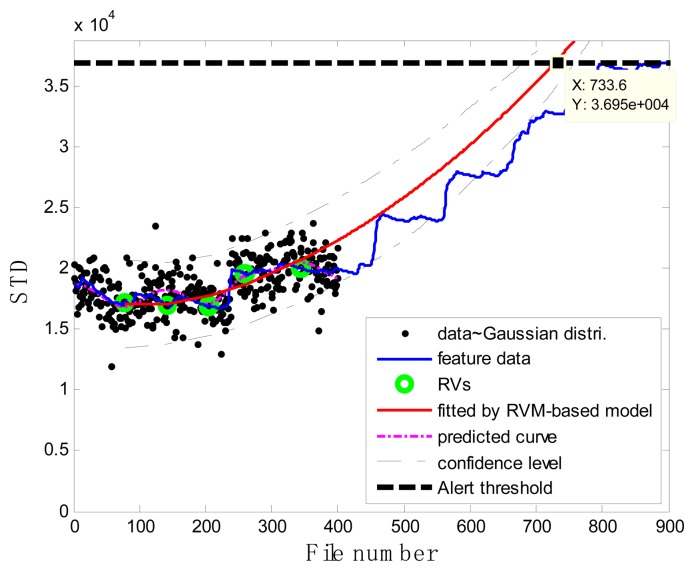
Inspection file number *x*_400_ = 400.

**Figure 11.**
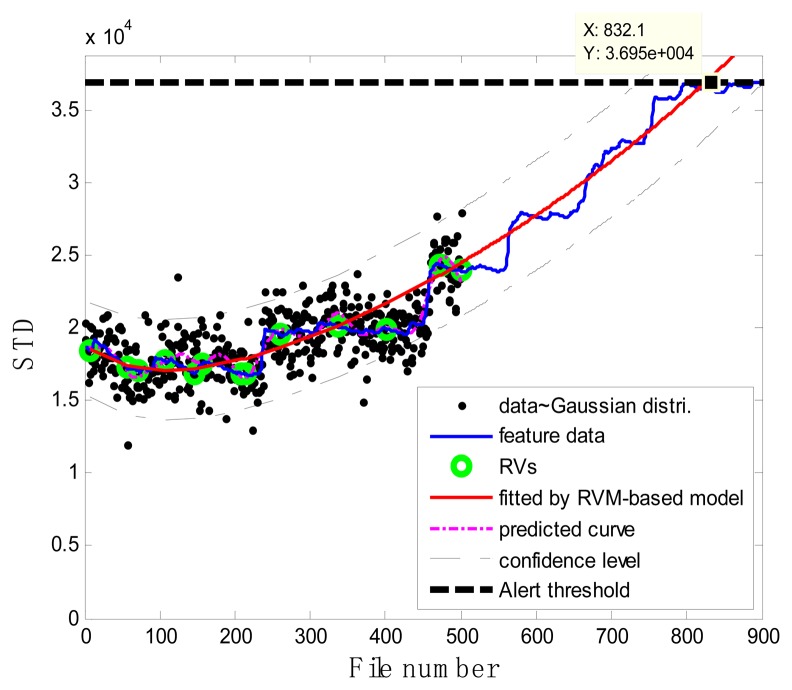
Inspection file number *x*_500_ = 500.

**Figure 12.**
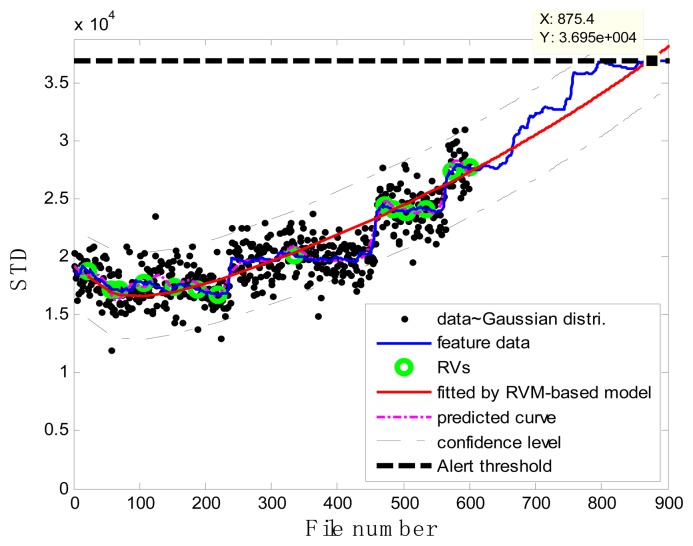
Inspection file number *x*_600_ = 600.

**Figure 13.**
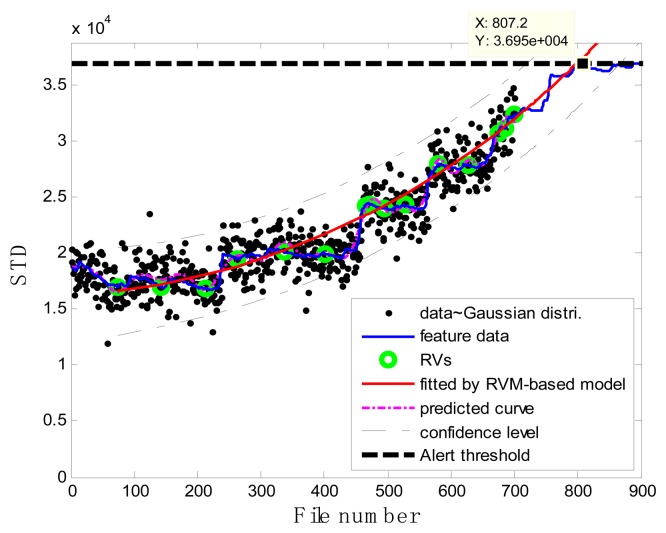
Inspection file number *x*_700_ = 700.

In the case of T2G1C3, the vibration signals were sampled from the Suction Pipe. During the feature extraction phase, the sliding window width was selected as 5. The data contained in the first 100 files were taken to represent the steady state of the impeller. The feature extraction results are plotted in Figure 6. The alert threshold was set equal to the maximum STD value, and thus the file number at the corresponding intersected point could be easily obtained, *i.e.*, 890. The prognostic results for T2G1C3 at the inspection file number *x_j_* ={200,300,400,500,600,700} are presented in Figures 8, 9, 10, 11, 12 and 13.

**Figure 14.**
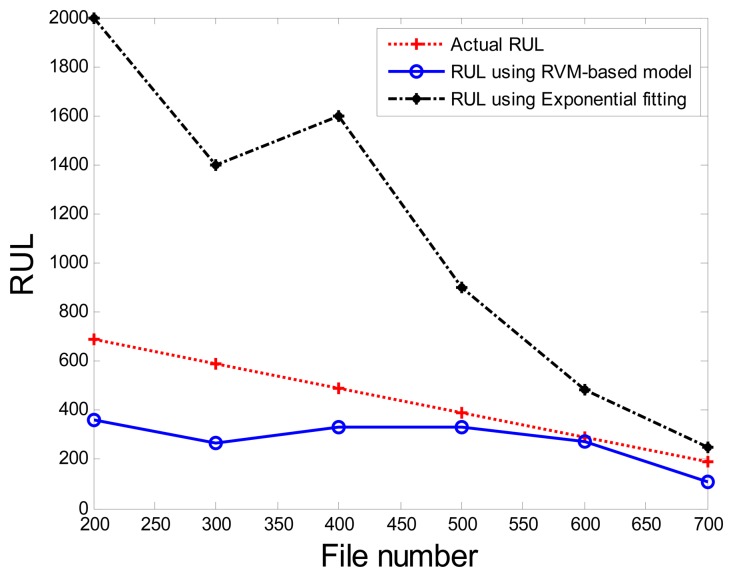
Comparison of prognosis performance using the RVM-based model and exponential fitting for dataset **T2G1C3**.

Comparisons of the results for datasets **T2G1C3** are shown in Table 2. Because the exponential fitting cannot provide information on the confidence bounds of predictions, the comparison results can only be described by the mean value of the estimated RUL at each inspection file number. Moreover, the comparison results are plotted in Figure 14 in order to clearly demonstrate the performance of the developed procedure. From Table 2, it is apparent that the RVM-based model outperforms the exponential fitting. During the service stage of impeller, compared to the exponential fitting, the RVM-based model does not yield any overestimation of RUL, a factor that is very appealing for an engineer planning the maintenance strategy for the pumps.

### Case 2: T2G1C4

**Figure 15.**
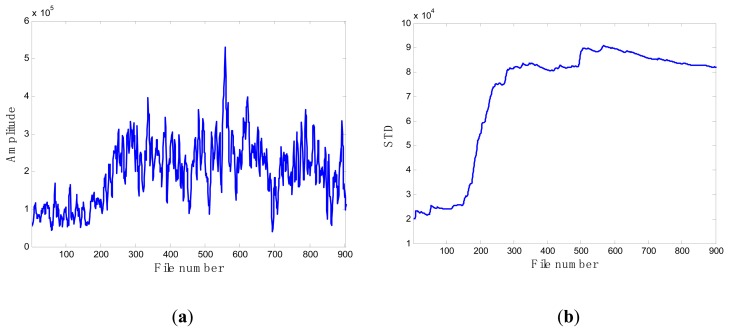
(**a**) Energy evolution (T2G1C4) (**b**) The standard deviation values (T2G1C4).

**Figure 16.**
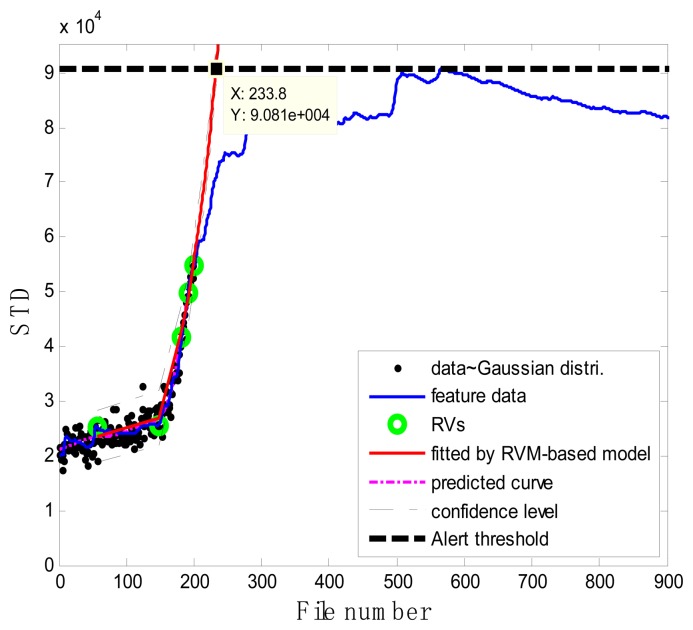
Inspection file number *x*_200_ = 200.

**Figure 17.**
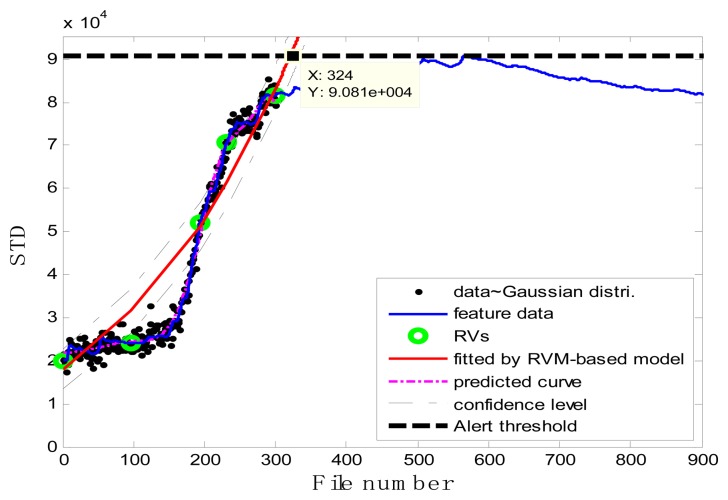
Inspection file number *x*_300_ = 300.

**Figure 18.**
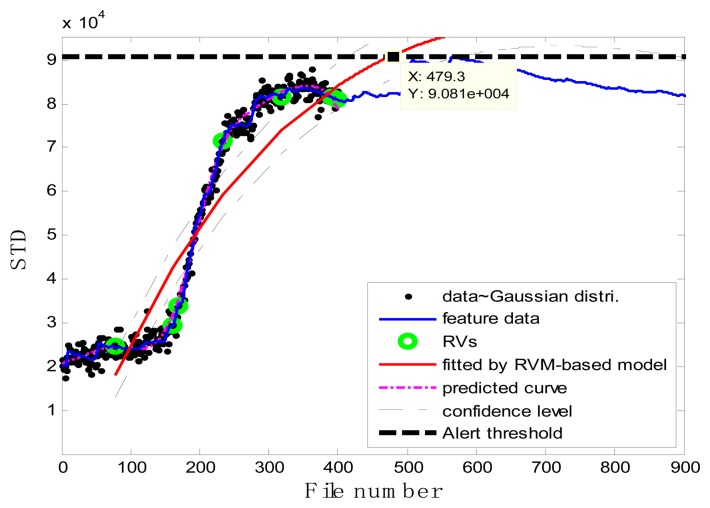
Inspection file number *x*_400_ = 400.

**Figure 19.**
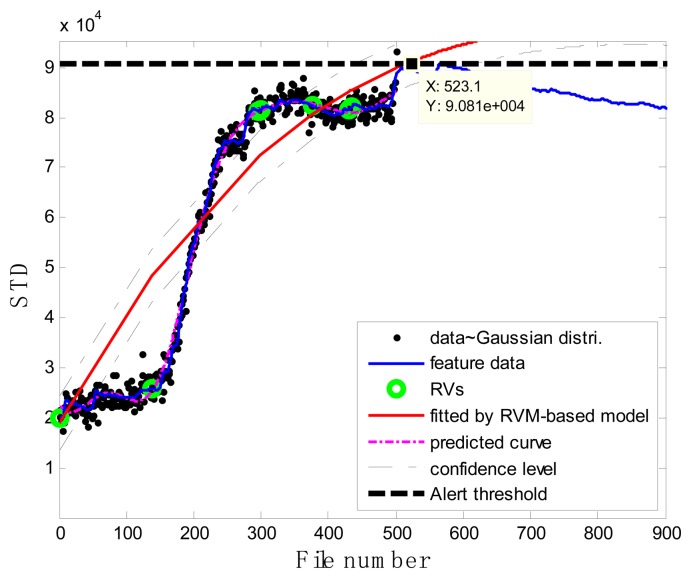
Inspection file number *x*_500_ = 500.

As shown in Figure 15, in the case of T2G1C4, the energy evolution presents an increasing trend until the file number equals 568, after which a slightly decreasing trend follows. An explanation for this behavior is as follows. The pump impeller is usually the first suffered component and wears out faster than other components. As the slurry pump undergoes continuous operation with oil sands, the number of crack occurred in the impeller's vanes increases. The rough surfaces of cracks will be easier to hook sands and small rocks, causing more cracks to occur [see Figure 3b(ii)]. Hence, the surfaces of the vanes become rougher and rougher, thus causing the continuous increase of vibration energy. Therefore, in the temporal plot of vibration energy as shown in Figure 15a, one will see the vibration energy level jumping up and down more. The surfaces of the vanes will become smoother due to the continuation of encountering the water or liquid. So the variation of vibration energy level began to decrease slightly instead. The alert threshold was therefore set at the corresponding STD value when the file number was equal to 568. For the feature extraction, the sliding window width was also selected as 5. The data during the first 60 file numbers were taken to be the steady state of the impeller as shown in Figure 15a. The prognostic results for T2G1C4 at the inspection file number *x_j_* = {200,300,400,500} are presented in Figures 16, 17, 18 and 19.

**Figure 20.**
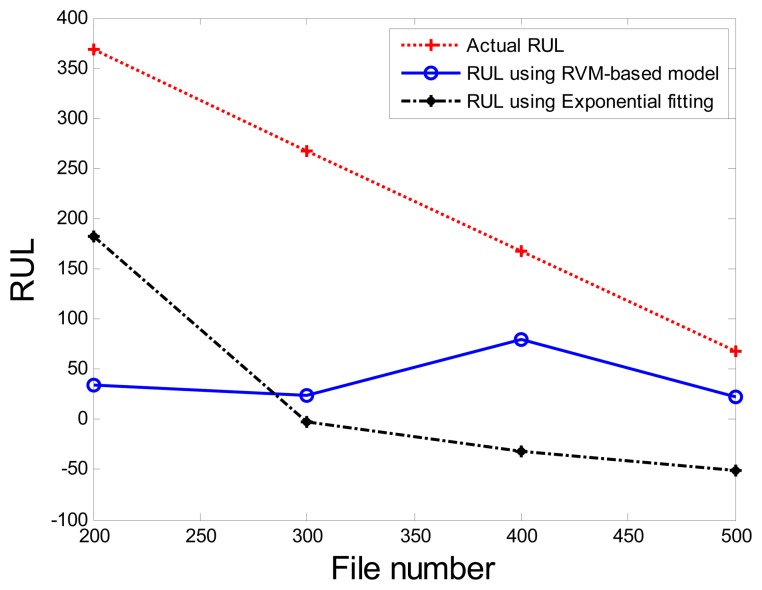
Comparison of prognosis performance using the RVM-based model and exponential fitting for dataset **T2G1C4**.

Correspondingly, the comparisons of the results for datasets **T2G1C4** are shown in Table 3. More clear comparison of the prognosis performance using the RVM-based model and exponential fitting for dataset T2G1C4 is plotted in Figure 20. From Table 3, it is clear that the RVM-based model generally outperformed the exponential fitting, especially through not presenting any underestimation compared with the exponential fitting. Additionally, it is shown than the prognosis accuracy presents better in the later service stage of impeller compared with itself in the early service stage from Figure 20, although the prognosis accuracy is still not as expected. Overall, the RVM-based model yielded results superior to those of the exponential fitting in terms of RUL prognosis. A particular empirically observed feature of the developed method, namely that it does not result in underestimation or overestimation during the whole working stage, is an advantage in practical applications for system maintenance scheduling to avoid cost waste and unexpected downtime.

The performance of the developed procedure for estimating the impeller RUL is further evaluated by using the weighted average accuracy of prediction. The weighted average accuracy of prediction may be calculated using the following formula [28]:

(22) $$\text{Weighted average of accuracy}=\frac{\sum_{j}\omega_{j}*(1-\frac{\left|RUL_{A}(x_{j})-R\hat{U}L(x_{j})\right|}{RUL_{A}(x_{j})})*100\%}{\sum_{j}\omega_{j}}.$$

The weights *ω_j_* are directly proportional to the inspection file number *x_j_*. Taking the dataset of T2G1C3 for example, *ω*_200_ = 200/2700 = 0.0741, *ω*_300_ = 200/2700 = 0.1111 Note that late predictions were penalized more heavily than early predictions. Hence, the designed performance indicator corresponded with actual needs and was more trustworthy in practical applications. The results with the weighted accuracy of prediction from the above applications are summarized in Table 4.

From the results listed in Table 4, it is evident that the RVM-based model yielded better prediction accuracy compared with that based on exponential fitting. However, it should be noted that the data used in this study were collected from an oil sand pump operating in the field and, hence, were unavoidably contaminated by many uncertain factors (such as disturbances from the hybrid fluid-solid material circulating through the impeller outlet until discharge) arising from the extremely complicated working conditions. A certain unevenness in the performance of any predictive model is therefore to be expected. Also, it is worth noting that only a single model (RVM) was adopted to train the prediction system (choosing the useful RVs). This might not have been sufficient to provide a completely robust solution in such a rugged working environment as that of the oil sand pumps.

## 5. Conclusions

This paper has presented a model combining relevance vector machines (RVMs) and a sum of two exponential functions that can be used for pump impeller prognosis and for the estimation of the pump's remaining useful life (RUL). The data used in the case study were all sampled from the field, *i.e.*, a pump in actual use in the oil industry. It should be noted that the field datasets were used for pump prognosis for the first time in a way that is much more challenging in terms of data pre-processing, feature extracting, and model building than in an analysis based on datasets collected in laboratory conditions. To solve the non-stationary problem emerging from the vibration data, a novel feature extracting process was proposed to arrive at a feature that increased generally as damage developed in the pump impellers. Different alert threshold levels were set for the two datasets sampled from two different positions on the same pump dependent on the practical degradation trends extracted from the datasets.

The proposed procedure was found to be capable of treating degradation signals for RUL estimation and yielded better performance than conventional standalone exponential fitting. However, owing to the extremely complicated running environment of the field pump, the weighted average accuracy of the prediction was not as high as expected. There is certainly room for improvement, and the authors propose to devote their future research efforts to the development of novel ensemble prognostic models that can further improve the predictive accuracy of this model.

## Figures and Tables

**Table 1.** Goodness of fit statistics used for comparing three functions.

**Goodness of Fit**	**R-square**	**Adjusted R-square**	**RMSE**
Sum of two exponential functions	0.9149	0.9147	0.1348
One exponential function	0.4874	0.4869	0.3305
Quadratic polynomial	0.8688	0.8686	0.1673

**Table 2.** Values of *RÛL*(*x_j_*) using the RVM-based model and exponential fitting.

**Inspection File Number** (*x_j_*)	**Actual** *RUL_A_*(*x_j_*)	*RÛL*(*x_j_*) **Using RVM-based Model**	*RÛL*(*x_j_*) **Using Exponential Fitting**
200	690	359.4	2,000
300	590	268.7	1,400
400	490	333.6	1,600
500	390	332.1	901
600	290	275.4	483
700	190	107.2	249

**Table 3.** Values of *RÛL*(*x_j_*) using the RVM-based model and exponential fitting.

**Inspection File Number** (*x_j_*)	**Actual** *RUL_A_*(*x_j_*)	*RÛL*(*x_j_*) **Using RVM-Based Model**	*RÛL*(*x_j_*) **Using Exponential Fitting**
200	368	33.8	181.6
300	268	24	−2.9
400	168	79.3	−32.2
500	68	23.1	−50.5

**Table 4.** The weighted average accuracy of the prediction for pump impeller.

**Weighted Average Accuracy of Prediction**			

**T2G1C3**		**T2G1C4**	
RVM-based model	Exponential fitting	RVM-based model	Exponential fitting
70.51 %	25.31 %	28.85%	7.05%
